# *ALDH2* variance in disease and populations

**DOI:** 10.1242/dmm.049601

**Published:** 2022-06-24

**Authors:** Che-Hong Chen, Benjamin R. Kraemer, Daria Mochly-Rosen

**Affiliations:** Department of Chemical and Systems Biology, Stanford University School of Medicine, Stanford, CA 94305, USA

## Abstract

The ALDH2*2 missense variant that commonly causes alcohol flushing reactions is the single genetic polymorphism associated with the largest number of traits in humans. The dysfunctional *ALDH2* variant affects nearly 8% of the world population and is highly concentrated among East Asians. Carriers of the ALDH2*2 variant commonly present alterations in a number of blood biomarkers, clinical measurements, biometrics, drug prescriptions, dietary habits and lifestyle behaviors, and they are also more susceptible to aldehyde-associated diseases, such as cancer and cardiovascular disease. However, the interaction between alcohol and ALDH2-related pathology is not clearly delineated. Furthermore, genetic evidence indicates that the ALDH2*2 variant has been favorably selected for in the past 2000-3000 years. It is therefore necessary to consider the disease risk and mechanism associated with ALDH2 deficiency, and to understand the possible beneficial or protective effect conferred by ALDH2 deficiency and whether the pleiotropic effects of *ALDH2* variance are all mediated by alcohol use.

## Abstract (precis)

**Summary:** The alcohol flushing-associated ALDH2*2 variant is one of the most common genetic polymorphisms in humans. This variant exhibits complex pleiotropic effects associated with alcohol consumption and aldehyde toxicity, which diversely impact human health.

## Introduction

Mitochondrial aldehyde dehydrogenase 2 (ALDH2) deficiency is one of the most common enzymopathies in human populations. One prominent East Asian-specific missense variant, E504K [single-nucleotide polymorphism (SNP) ID rs671, G>A] or the ALDH2*2 allele, affects an estimated 540 million East Asians, or 8% of the world population (Brooks et al., 2009). ALDH2 plays an important function in detoxification of acetaldehyde, a direct metabolite of alcohol generated by alcohol dehydrogenase (ADH) (Zakhari, 2006). ALDH2 metabolizes other reactive and oxidative stress-generated aldehydes, such as 4-hydroxynonenal (4-HNE) formed by lipid oxidation, that are associated with human diseases (Chen et al., 2014; Gross et al., 2015). In 1981, Harada et al. first demonstrated the relationship between ALDH2 deficiency, elevated blood acetaldehyde levels and alcohol flushing reactions, with symptoms of facial flushing, palpitation, tachycardia, muscle weakness, headache and nausea presenting in nearly 43% of the Japanese cohort of his study (Harada et al., 1981; Brooks et al., 2009). This phenomenon is now well documented.

The spectrum of different diseases associated with ALDH2 deficiency and aldehyde toxicity is well covered by many recent reviews, and includes a higher risk of cancer and cardiovascular disease (Gross et al., 2015; Chen et al., 2014, 2016, 2019; Chang et al., 2017; Wang et al., 2021; Kimura et al., 2019; Zhao and Wang, 2015). Both alcohol and its metabolite acetaldehyde have been classified as Group 1 human carcinogens by the World Health Organization (WHO) International Agency for Research on Cancer (IARC) since 2007 due to acetaldehyde's reactivity and genotoxicity to DNA, proteins and macromolecules (Baan et al., 2007; IARC Working Group on the Evaluation of Carcinogenic Risks to Humans, 2010). More specifically, epidemiological studies have now demonstrated that alcohol is causally related to malignant tumors of the oral cavity, pharynx, larynx, esophagus, liver, colorectum and female breast. Indeed, East Asians carrying the ALDH2*2 variant are at higher risk of esophageal, head and neck, and lung cancer if they regularly consume alcohol compared to people with the wild-type *ALDH2* (Baan et al., 2007; IARC Working Group on the Evaluation of Carcinogenic Risks to Humans, 2010; Im et al., 2022).

Owing to the increasing scope of ALDH2*2 association with disease, significant interest and research effort have been devoted to targeting ALDH2 in disease prevention and treatment (Gao et al., 2022; Zhang and Fu, 2021; Kimura et al., 2019; Chen et al., 2014; Lee et al., 2021). Small-molecule ALDH2 enzyme activators, such as Alda-1 and Alda-64, that can enhance the wild-type ALDH2 activity and restore the structure and function of the ALDH2*2 variant enzyme have been reported and, if effective in humans, could reduce the risk of developing associated disease (Chen et al., 2008; Perez-Miller et al., 2010; Chen et al., 2016). The efficacy of Alda-1 in enhancing ALDH2 activity and detoxification of reactive aldehydes has been reported in different cellular and animal models of diseases (Münzel and Daiber, 2018; Mittal et al., 2020; Kimura et al., 2019). Therapeutic agents based on ALDH2 activators such as Alda-1 and Alda-64, therefore, deserve future clinical characterization and development for diseases that could be treated by enhancing or restoring ALDH2 enzyme activity. In this Perspective, we will explore the role of the ALDH2*2 variant in human disease and health and assess its role beyond alcohol metabolism and related diseases.

“The majority of ALDH2*2 variant carriers still do not recognize the health risks associated with combining ALDH2 deficiency and alcohol consumption […] this highlights the importance of public health campaigns to inform the affected population.”

## Bioinformatics and pleiotropic effect of *ALDH2* variance in East Asian populations

**Fig. 1.**
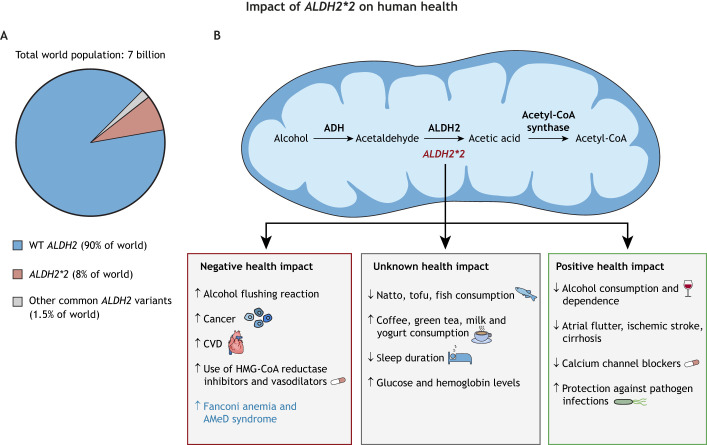
Distribution of ALDH2 variants in the human population and the impact of ALDH2*2 on human health outcomes. (A) The pie chart displays the prevalence of common *ALDH2* variants that cause decreased enzyme activity in East Asian and non-East Asian human populations. (B) The mitochondria schematic shows alcohol metabolism and displays the effects of ALDH2*2 on health. Negative impacts are often mediated by alcohol consumption due to accumulation of acetaldehyde; those in blue font are not mediated by alcohol consumption. ADH, alcohol dehydrogenase; ALDH2, aldehyde dehydrogenase 2; AMeD, aplastic anemia, mental retardation and dwarfism; CVD, cardiovascular disease; HMG-CoA, 3-hydroxy-3-methylglutaryl coenzyme A; WT, wild type.

Big data and bioinformatic analyses of *ALDH2* variance using large human multi-omics databases, hospital clinical data and questionnaire surveys of diet and lifestyle have become an important tool for basic and translational population research. Recent bioinformatic data from BioBank Japan (BBJ) provide the most comprehensive association landscape between the ALDH2*2 variant and human phenotypes in the Japanese population (Sakaue et al., 2021). The BBJ project conducted phenome-wide association studies (PheWAS) and genome-wide association studies (GWAS) correlating genetic data with electronic medical records of 159 different diseases, 38 biomarkers and 23 medication usage reports among 179,000 Japanese patients. Among the 220 analyzed human phenotypes, the ALDH2*2 missense variant stood out as the most significant functional SNP that was associated with the largest number of different human traits from the genomes of this Japanese cohort. The pleiotropic effect of ALDH2*2 was demonstrated by its association with 47 human phenotypes, including a positive correlation with esophageal cancer, cardiovascular diseases (angina, myocardial infarction), prescription drug use of vasodilators and 3-hydroxy-3-methylglutaryl coenzyme A (HMG-CoA) reductase inhibitors, and a negative correlation with cirrhosis, atrial flutter, ischemic stroke, blood glucose level, blood pressure, uric acid, gamma-glutamyl transferase (GGT), mean corpuscular volume, mean corpuscular hemoglobin and prescription drug use of calcium channel blockers (Fig. 1) (Sakaue et al., 2021). The effect of alcohol consumption on the relationship between phenotype and genotype is unknown, because alcohol consumption data were not included in this study. In a separate study, Hu et al. found that the ALDH2*2 variant was associated with 13 out of 29 blood biochemical markers (e.g. glucose, uric acid) in a study consisting of 1999 male subjects in Southern China (Hu et al., 2019). Many other significant associations between the ALDH2*2 variant and human traits have been demonstrated in other GWAS of the East Asian populations. They included a negative correlation with gout (Sakiyama et al., 2017) and alcohol use disorder (Zhou et al., 2022; Jorgenson et al., 2017), and a positive correlation with metabolic syndrome (Wang et al., 2016b), obesity (Wang et al., 2016b) and body mass index (Wen et al., 2014). In addition to having a strong negative influence on the quantity of alcohol consumption, the ALDH2*2 variant affects other dietary habits. ALDH2*2 was found to be negatively correlated with the dietary intake of natto, tofu and fish, and positively correlated with the intake of coffee, green tea, milk and yogurt (Fig. 1) (Nakagawa-Senda et al., 2018; Matoba et al., 2020), among the Japanese population (Suzuki et al., 2021; Igarashi et al., 2019; Matoba et al., 2020), while no association with meat intake was found (Nakamura et al., 2021). A study involving 31,230 Japanese individuals also confirmed the influence of the ALDH2*2 variant on sleep pattern and duration, as ALDH2*2 carriers had shorter sleep durations compared to *ALDH2* wild-type individuals. Furthermore, this difference seemed to be amplified by alcohol consumption (Fig. 1) (Nishiyama et al., 2019). Although the mechanisms behind ALDH2*2 association with dietary habits beyond alcohol are unclear, changes in nutrient metabolism in ALDH2*2 carriers may contribute to human health and disease progression, which warrants further investigation.

**Figure:**
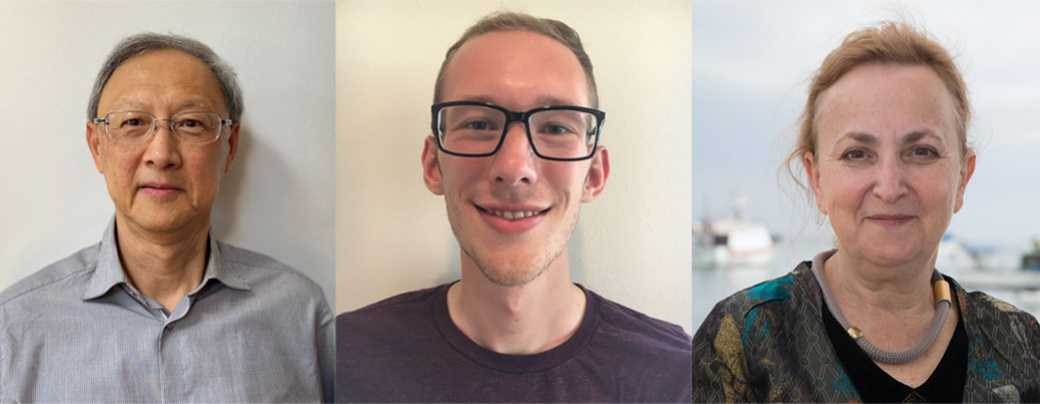
Che-Hong Chen, Benjamin R. Kraemer and Daria Mochly-Rosen (from left to right)

Some of the challenges in studying the effect of *ALDH2* variance in populations are ascertaining the causal relationship between alcohol consumption and a particular phenotype, and understanding the complex interaction between the genetic influence of *ALDH2* polymorphism and the behavior of alcohol consumption. It is well known that the ALDH2*2 variant can lead to alcohol avoidance due to the unpleasant alcohol flushing response (Edenberg et al., 2019). However, alcohol aversion can be overcome by cultural and social factors (Brooks et al., 2009). Many heterozygous *ALDH2* variant carriers have developed tolerance to the acetaldehyde-induced flushing response and become habitual heavy drinkers. The number of alcoholics or heavy drinkers (consuming >400 g of ethanol per week) with the heterozygous ALDH2*2 genotype has risen rapidly in the past few decades among East Asians from 2-3% to 17-26% in Japan and Taiwan (Higuchi et al., 1994; Yokoyama et al., 2002; Chen et al., 1999). This subgroup of ALDH2*2 heterozygous alcohol drinkers is at higher risk of many alcohol-related diseases compared to ALDH2*2 heterozygous individuals who avoid alcohol consumption.

Im et al. conducted a prospective study following a cohort of 150,772 Chinese adults in ten different regions for 11 years, recording their *ALDH2* genetic information, alcohol consumption habits and risk of cancer development (Im et al., 2022). This large-scale genetic study utilizing the China Kadoorie Biobank (CKB) data provided the strongest direct causal relationship and interaction between alcohol use, cancer and *ALDH2* genotype. Carriers of the ALDH2*2 allele were associated with reduced alcohol consumption and overall alcohol-related cancers. However, among the group of alcohol drinkers who overcame the aversion to the alcohol flushing reaction, there was an alarmingly high and dose-dependent risk of alcohol-related cancers, especially upper aero-digestive tract cancers (Im et al., 2022). In another study involving 500,000 men and women in China, the strong predictive value of *ALDH2* genotypes on quantity of alcohol intake has been used to evaluate the effect of alcohol consumption on cardiovascular disease using the tool of Mendelian randomization (Millwood et al., 2019). In contrast to the protective effects of moderate alcohol consumption against stroke and coronary heart diseases based on conventional epidemiology (Ronksley et al., 2011; Roerecke and Rehm, 2012; Larsson et al., 2016), genetic epidemiology showed that the apparent protective effect against stroke and myocardial infarction does not exist in all participants of this study (Millwood et al., 2019). Even low alcohol intake was strongly and positively associated with increased incidences of ischemic stroke, hemorrhagic stroke and higher blood pressure in all participants of this study (Millwood et al., 2019).

Thus far, alcohol consumption data, including frequency and quantity, are not readily available in many published human bioinformatics analyses, clinical studies and database collections. This information is critical and should be included in future population-based studies involving ALDH2 deficiency and human disease, and potentially along with other related genetic loci such as ADH alleles. Integration of all necessary data will become increasingly valuable for verification of therapeutic targets, drug design and prevention strategies related to East Asian health issues. In addition, an increasing trend of alcohol consumption and alcohol-related disease burden has been observed in East Asian populations in recent years (Manthey et al., 2019; Rumgay et al., 2021). Given that the majority of ALDH2*2 variant carriers still do not recognize the health risks associated with combining ALDH2 deficiency and alcohol consumption (Brooks et al., 2009; Newman et al., 2013), this highlights the importance of public health campaigns to inform the affected population about alcohol flushing syndrome and its alcohol-related health risks (Newman et al., 2017; Hendershot et al., 2010).

“Although the ALDH2*2 missense mutation is the predominant dysfunctional variant affecting an estimated 540 million East Asians, other missense variants of the *ALDH2* gene are found in the human population.”

## Alternative *ALDH2* variants in global populations

Alcohol flushing syndrome has also been reported in non-East Asian ethnic groups (Fig. 1) (Linneberg et al., 2010). Although the ALDH2*2 missense mutation is the predominant dysfunctional variant affecting an estimated 540 million East Asians, other missense variants of the *ALDH2* gene are found in the human population. Chen et al. compiled five additional common *ALDH2* missense variants with allele frequencies ranging from 0.6% to 2.5% in groups of African, Latino, South Asian and Finnish genetic ancestries (Chen et al., 2020). *In vitro* and cell-based studies showed that these additional missense variants also result in reduced ALDH2 enzymatic activity. Thus, there may be an estimated 120 million people of non-East Asian genetic ancestry, representing 1.5% of the world population, harboring reduced ALDH2 activity (Chen et al., 2020). The association of these variants with human disease has yet to be explored, but due to the decreased activity it is likely that these patients may be at risk for related diseases similar to ALDH2*2 variant carriers, such as cancer, cardiovascular disease and others mentioned in this article. The clinical significance of these additional and common *ALDH2* missense variants should be explored further, as these sizeable subpopulations may also be at increased risk for the many diseases associated with ALDH2*2, especially if alcohol drinking is prevalent in those communities.

“Aldehyde toxicity can not only be produced from alcohol consumption directly, but can also come from endogenous sources that trigger or accelerate disease progression from a non-alcohol source in patients with ALDH2 deficiency.”

## ALDH2 metabolism and disease beyond alcohol

Individuals carrying the ALDH2*2 variant are clearly more susceptible to diseases caused by the toxic effect of acetaldehyde from alcohol drinking. However, there are also non-alcohol related diseases that ALDH2*2 carriers are more prone to due to environmental exposure or endogenous aldehyde production (Fig. 1). Fanconi anemia is a rare disease caused by mutations in DNA repair genes and characterized by bone marrow failure, developmental abnormalities and multiple cancer predisposition (Auerbach, 2009; Kee and D'Andrea, 2012). Evidence has shown that naturally occurring and endogenous genotoxic aldehydes, such as formaldehyde and acetaldehyde, could be primary sources of DNA damage that induce genome instability in hemopoietic stem cells in Fanconi anemia patients (Wang et al., 2022; Garaycoechea and Patel, 2014; Brooks and Zakhari, 2014). Hira et al. showed that ALDH2 deficiency accelerated bone marrow failure in pediatric patients with Fanconi anemia (Hira et al., 2013). Malformations in multiple anatomic locations were observed more frequently in ALDH2-deficient patients diagnosed with Fanconi anemia in Japanese patients (Hira et al., 2013). In addition, ALDH2*2 has recently been identified, together with homozygous recessive mutations in one of the alcohol dehydrogenase genes (*ADH5*), for a digenic disease called aplastic anemia, mental retardation and dwarfism (AMeD) syndrome due to impaired formaldehyde clearance in Japanese patients (Oka et al., 2020). This disease presents etiology and clinical features similar to Fanconi anemia, but patients do not have any detectable mutations in genes involved in the Fanconi anemia DNA repair pathway. These diseases highlight how aldehyde toxicity can not only be produced from alcohol consumption directly, but can also come from endogenous sources that trigger or accelerate disease progression from a non-alcohol source in patients with ALDH2 deficiency.

## Is ALDH2 deficiency all bad?

The rapid spread and enrichment of the East Asian ALDH2*2 variant within the past 2000-3000 years (∼100 generations) of human history is a mystery and implies a favorable biological selection of this dysfunctional aldehyde metabolizing mutation (Luo et al., 2009; Field et al., 2016). Singleton density score was used to estimate changes in allele frequency by natural selection in the human genome across this time period (Field et al., 2016; Wang et al., 2016a). Applying this method to whole-genome sequencing and GWAS data of >170,000 individuals from the BBJ project identified a strong selection signature in both alcohol dehydrogenase 1B (*ADH1B*; 4q23), which encodes the ADH1B protein that oxidizes ethanol, and *ALDH2* (12q24) chromosome regions (Okada et al., 2018). This study confirmed that ALDH2*2 indeed underwent rapid change in its allele frequency in the past 2000-3000 years, implying that the simultaneous positive selection of both the *ADH1B* rs1229984 and ALDH2*2 variants in the Japanese population was tied to traits or diseases that were mediated by alcohol consumption or nutrition metabolism (Okada et al., 2018). In support of this positive selection, Sakause et. al. reported a protective effect of ALDH2*2 on atrial flutter, ischemic stroke, cirrhosis and reduced use of calcium channel blockers, as mentioned previously (Sakaue et al., 2021). Interestingly, ALDH2 deficiency has also been reported to confer protection against tuberculosis infection, owing to the increased aldehydic load of ALDH2*2 carriers (Park et al., 2014). Furthermore, a strong geographic correlation between ALDH2*2 and hepatitis B viral infection in East Asia has been observed (Lin and Cheng, 2002). This led to a hypothesis that the aversion to alcohol in ALDH2*2 carriers resulted in a longer life span due to protection against hepatitis B infection, liver cirrhosis and liver cancer (Lin and Cheng, 2002). As discussed in previous sections, the unpleasant alcohol flushing reaction caused by the ALDH2*2 variant offers its carriers a strong, but not impenetrable, protection against alcohol abuse and alcohol-related organ damage and diseases. The rapid increase in the ALDH2*2 variant in East Asia has been proposed to coincide with the period of agricultural domestication of rice in China (Zhu et al., 2021; Wang et al., 2016a). Therefore, is alcohol metabolism or nutritional adaptation the major biological driving force for the selection of the ALDH2*2 variant? Or could it be possible that the rise in ALDH2*2 was due to its protection against a widespread and serious infectious disease in East Asia such as tuberculosis or hepatitis infection? Alternatively, the ALDH2*2 variant may be favored in a specific condition under which the interaction between *ALDH2* genotype and alcohol consumption modulated the fitness or outcome of a common disease. These are the questions that remain to be answered to explain why ALDH2*2 has become such a prevalent genetic variant in human populations.

“Although it is clear that the ALDH2*2 variant allele is tied to an elevated health risk of many disease and disease-related traits, there are other diseases and phenotypes upon which the ALDH2 variant appears to have a protective or beneficial effect.”

## The future of ALDH2 research

Although it is clear that the ALDH2*2 variant allele is tied to an elevated health risk of many disease and disease-related traits, there are other diseases and phenotypes upon which the *ALDH2* variant appears to have a protective or beneficial effect, such as atrial flutter, ischemic stroke, cirrhosis and reduced use of calcium channel blockers. Recently published research has discovered new *ALDH2* variant-associated human traits, such as dietary preferences and sleep duration, of which the physiological impact or the net effect on human wellbeing are not yet known. Furthermore, are all the associated positive or negative effects on human health entirely mediated by alcohol and aldehyde toxicity? Or does the *ALDH2* variant have some inherent advantage on human fitness that is independent of alcohol metabolism? The focus of future research into human ALDH2 variant phenotypes should be understanding the exact molecular mechanisms, the biological effect of different species of aldehydes, and both external and internal factors that together may affect human health and disease. A deeper understanding of the interactions between *ALDH2* genotype, phenotype and environment will also be beneficial and can serve as the blueprint for studies of other common human genetic variants that play a role in human disease and metabolism.
